# Managing high-risk patients: the Mass General care management programme

**DOI:** 10.5334/ijic.2245

**Published:** 2015-09-23

**Authors:** Dennis L. Kodner

**Affiliations:** Of Integrated Care Group, LLP; Of Medicine in the Division of Geriatric Medicine at McGill University, Montreal, Canada

**Keywords:** co-ordinated care, case management, physician practice-based models, high-cost patients, multiple chronic conditions, multiple hospitalisations

## Abstract

The Massachusetts General Care Management Program (Mass General CMP or CMP) was designed as a federally supported demonstration to test the impact of intensive, practice-based care management on high-cost Medicare fee-for-service (FFS) beneficiaries—primarily older persons—with multiple hospitalisations and multiple chronic conditions. The Massachusetts General Care Management Program operated over a 6-year period in two phases (3 years each). It started during the first phase at Massachusetts General Hospital, a major academic medical centre in Boston, Massachusetts in collaboration with Massachusetts General Physicians Organisation. During the second phase, the programme expanded to two more affiliated sites in and around the Boston area, including a community hospital, as well as incorporated several modifications primarily focused on the management of transitions to post-acute care in skilled nursing facilities. At the close of the demonstration in July 2012, Mass General Massachusetts General Care Management Program became a component of a new Pioneer accountable care organisation (ACO). The Massachusetts General Care Management Program is focused on individuals meeting defined eligibility criteria who are offered care that is integrated by a case manager embedded in a primary care practice. The demonstration project showed substantial cost savings compared to fee-for-service patients served in the traditional Medicare system but no impact on hospital readmissions. The Massachusetts General Care Management Program does not rest upon a “whole systems” approach to integrated care. It is an excellent example of how an innovative care co-ordination programme can be implemented in an existing health-care organisation without making fundamental changes in its underlying structure or the way in which direct patient care services are paid for. The accountable care organisation version of the Massachusetts General Care Management Program includes the staffing structure, standards of practice, collaborative approach to care transitions and information technology tools that were used in the original demonstration project.

## The model of integrated care

### Background

In the United States, care co-ordination is widely recognised as a critical component of publicly- and privately funded health care for the clinical management of high risk, complex and costly patients whose needs cut across multiple providers, services and settings within a system that is largely considered fragmented and uncoordinated. While integrated delivery systems (e.g., Kaiser Permanente and Group Health Puget Sound) and special “carve out” managed care programmes (e.g., PACE) operating in budgeted or capitated environments have been especially successful in improving quality and reducing costs through the use of care management and other co-ordinated care approaches, research from the traditional fee-for-service^1^ sector is mixed. Since fee-for-service remains the dominant form of payment for physician and other health-care services in the United States and care co-ordination services play an important role in new “medical home”^2^ and “accountable care organisation”^3^ models, finding care co-ordination models that work in the world of open-ended, “pay-as-you-go” care has become a vital concern. This is the challenging context in which the Massachusetts General Care Management Program (Mass General CMP or CMP) was developed.

Launched in August 2006, the Massachusetts General Care Management Program was designed to provide intensive, practice-based care management (PBCM) to high-cost Medicare^4^ fee-for-service beneficiaries. The original 3-year demonstration project, which has recently completed its second 3-year phase, is co-sponsored by Mass General (also referred to as MGH), a 900-bed general hospital and teaching affiliate of Harvard Medical School and Massachusetts General Physicians Organization (MGPO), a multi-specialty physician network in Boston, MA. Both organisations are founding members of Partners Healthcare, an integrated delivery system which includes two academic medical centres, community hospitals, specialty hospitals, community health centres, a physician network, home health and long-term care services and other health-care-related entities. The demonstration programme operates under a contract with the federal government’s Centers for Medicare and Medicaid (CMS)^5^ as part of that agency’s Care Management for High Cost Beneficiaries (CMHCB) demonstration. The primary objective of the Care Management for High Cost Beneficiaries demonstration is to test new intervention strategies and a pay-for-performance (P4P)^6^ contracting model focused on Medicare beneficiaries who are high cost and/or have complex conditions.

During the initial 3-year phase of the Massachusetts General Care Management Program, which is the primary focus of this case study, eligible Medicare fee-for-service beneficiaries from several Massachusetts counties including Boston and surrounding areas and with a high level of disease severity were voluntarily enrolled in the programme. Nurse case managers were embedded in each of the Massachusetts General Physicians Organization primary care practices. They developed close one-on-one relationships with programme participants, co-ordinated care throughout the health-care continuum, acted as a communications hub between patients and providers, and delivered or arranged other important support services. The Massachusetts General Care Management Program is housed in the hospital’s Case Management Department. However, programme leadership operates within a highly matrixed organisational environment that cuts across Mass General and Massachusetts General Physicians Organization.

There are two main goals of Massachusetts General Care Management Program, namely, to improve: (1) quality of care and outcomes for Medicare beneficiaries and (2) quality of work life of primary care physicians and ultimately attract more physicians to the field. Overall, the programme is part of Partner Healthcare’s larger vision to restructure the model of primary care, enhance workflow and process improvement and encourage the delivery of evidence-based care.

The programme’s original 3-year demonstration period ended in June 2009 having served over 2,600 enrolled patients. Centers for Medicare and Medicaid commissioned an independent evaluator, Research Triangle Institute International (RTI), to evaluate the initial demonstration. Using a comparison research design, findings point to significant declines in both inpatient admissions and emergency department (ED) use rates, annual improvements in mortality, high patient and physician satisfaction, and impressive savings in annual Medicare costs.

Centers for Medicare and Medicaid announced in 2009 that because the Massachusetts General Care Management Program was so successful, the programme would be extended for another 3-year period until 31 July 2012.

### Client group

Massachusetts General Care Management Program, and the Care Management for High Cost Beneficiaries demonstration of which it is part, is designed to creatively address the following challenges inherent in the Medicare population:

- Patients with multiple chronic conditions are high users of Medicare-covered services and account for a disproportionately large share of total Medicare spending [1,2].
- Hospital admissions and readmissions for this group are largely responsible for these high Medicare costs [3].
- A substantial proportion of these Medicare hospitalisations are for conditions that could be more appropriately and better addressed by effective outpatient treatment [1].

Since the ambition of the Massachusetts General Care Management Program is to enhance care outcomes for the complex, costly patient population described above and also to achieve savings for Medicare, the programme begins by targeting patients who are high users of health-care services that could benefit the most from enrolment in a care management programme. A combination of historical cost data (based on Medicare claims) and Hierarchical Condition Category (HCC) scores are used to identify a cohort of high-cost patients resulting from multiple hospitalisations and multiple chronic conditions, e.g., coronary artery disease, congestive heart failure, chronic obstructive pulmonary disease and cognitive impairment.

The Hierarchical Condition Category risk adjustment model used by Massachusetts General Care Management Program was originally developed for Centers for Medicare and Medicaid as an actuarial tool to calculate Medicare payment rates for Medicare Advantage (MA) managed care plans and Prescription Drug Plans (PDPs) based on enrolee demographics and health status. Higher risk scores indicate greater burden of illness and the likelihood of higher costs.

At the start of the programme, claims data for Medicare patients (15,230) of all 19 Massachusetts General Physicians Organization primary care practices (190 internal medicine physicians) were entered in an analytical database which also applied several inclusion (eligibility) and exclusion criteria (Table 1).^7^ This resulted in a pool of 2619 of potential Massachusetts General Care Management Program participants.^8^ For evaluation purposes, a comparison group was selected using similar criteria from patients being seen by primary care physicians in medical groups affiliated with four other academic medical centres in the Boston area.

On average, the pool’s high-risk patients were 76 years old and primarily female (51%). A significant cohort (11%) was disabled Medicare beneficiaries under 65 years of age. In addition to averaging 3.4 acute care hospitalisations and 12.6 active medications per year respectively, health-care costs for these patients were substantial: average annual costs of $22,500 (excluding pharmaceuticals) and total costs of nearly $58 million in the year prior to enrolment [4. Indeed, Massachusetts General Physicians Organization primary care physicians who reviewed the list of eligible patients in their own panels concurred that they were the sickest, most complex and highest risk patients being cared for [5.

Enrolment in Massachusetts General Care Management Program is voluntary on the part of the patient. Initially, Massachusetts General Care Management Program outsourced the initial patient outreach and engagement functions to Health Dialog, a health-coaching company. However, programme managers noticed confusion and suspicion on the part of eligible patients; this was leading to lower than anticipated enrolment rates [6. After 2 months, these activities were transferred in-house to the case managers themselves.

Active outreach to eligible patients included a letter sent out on Centers for Medicare and Medicaid letterhead by Mass General introducing the demonstration; a welcome letter from the Massachusetts General Care Management Program Medical Director with more detailed information on the programme; follow-up contacts with potential enrolees by phone; and, face-to-face meetings in physician offices. In addition, many of the physician practices sent their own letters encouraging their patients’ participation in Massachusetts General Care Management Program. Case managers were directly responsible for patient enrolment. However, some primary care physicians enrolled patients during medical office visits with case manager involvement. The enrolment process lasted the first 6 months of programme operations. Massachusetts General Care Management Program was successful in enrolling 88% of its original pool of eligible patients and 84% of the refreshed population. The programme had over 2,600 enrolled patients during the first phase.

### Approach to care

At the heart of the Mass General programme is intensive practice-based care management. A nurse case manager is assigned to each enrolled patient. Case managers also work directly with a group of primary care physicians in their offices to manage the care of about 180–220 patients each.^9^ Primary care physicians received a token payment of $150 per patient per year as an incentive to help cover the time involved with the programme. Being embedded in a medical practice provided case managers with physical proximity to the primary care physicians with whom they would be closely working; it also put them at the centre of patient care activity and signalled that they were an important part of the clinical team. Case managers play multiple roles: they serve as a vital communications hub for patients and providers; collaborate with patients, families and providers to assess clinical and support needs and develop an appropriate plan of care; coordinate patient care activities; and, act as a point of continuity [5].

The Massachusetts General Care Management Program relies upon a team of very experienced and highly skilled case managers with backgrounds in outpatient, hospital, home health care and health insurance company settings. The case managers selected not only have extensive clinical expertise to provide the highly individualised care demanded by complex patients but also the capacity to develop trusting, respectful, long-term relationships with both patients and physicians. Case managers received intensive training in standards of practice, assessment tools, palliative care and advance care planning, disease management and health coaching. Moreover, new case management team members are assigned mentors to learn beside and shadow.

In contradistinction to previous care co-ordination programmes that failed in part because programme goals and interventions were not clearly specified, the Massachusetts General Care Management Program developed a focused, best practice-based set of case management roles, responsibilities and activities (Table 2). Further, a “mass customisation” approach was employed to run an efficient and effective programme capable of achieving cost savings as well as deliver a predictable, high-quality standard of care.^10^

A critical element of the Massachusetts General Care Management Program is the intensive, one-on-one relationship between the practice-based case managers and their patients. This occurs through periodic telephone calls (at least once every 4–6 months), in-person interactions at the physician’s office or when hospitalised, and home visits on an as-needed basis.

During the programme’s initial phase, a total of 12 case managers worked with 190 primary care physicians in 20 practices located throughout the project area. In addition, there was a designated, centrally located case manager to work with hospital discharges, as well as a social worker to oversee a telephone reassurance programme geared to patients affected by social isolation.

The case management team was directly supported by the Massachusetts General Care Management Program project manager, an administrative assistant, a community resource specialist to connect patients with home- and community-based services, and a patient financial counsellor to assist with insurance-related issues [6].^11^

To expand case manager knowledge and skills, they were required to attend three case reviews per month and a monthly continuing education session. Peer-based learning also played a major role in furthering the development of team members’ skills.

Access to a wide range of health information technology (HiT) tools enabled case managers to perform the many activities for which they are responsible. In addition to Mass General’s existing electronic medical record (EMR), and clinical messaging and email systems, case managers were also supported by Mass General’s case management system (MIDAS). All case assessments, care plans and interventions were logged on Mass General’s case management system. To the extent that patient care activities were occurring outside of Mass General, Massachusetts General Physicians Organization and other provider organisations in Partners Healthcare, case managers obtained, aggregated and recorded that information on the electronic medical record and case management systems as appropriate.

There are two main clinical interventions provided by Massachusetts General Care Management Program: (1) case management and (2) management of care transitions. Additional “wraparound” support resources were made available to address specialised patient needs in the areas of mental health, medication management and end-of-life care. These components are described as follows:

#### Case management

The case manager is responsible for first conducting a health risk assessment (HRA) and then a comprehensive assessment for each patient.

Conducted during the enrolment process, the health risk assessment used clinical judgement to assign each patient to one of the three risk categories: Priority I is the highest risk and Priority III is the lowest risk (Table 3). The patient’s risk level was determined by the case manager in consultation with the primary care physician. Such risk stratification enabled the case manager to focus attention and time on individuals with the highest risk and most complex needs; the typical caseload includes 75 Priority I patients at any time. The comprehensive patient assessment, which evaluates the unique needs of each programme participant, was performed during the patient’s first 3 months in the programme. The multi-dimensional tool was developed in-house by the Massachusetts General Care Management Program team and contains several externally validated instruments (Table 4). Finally, using information collected from the assessment, case managers developed and implemented a care plan for each patient in conjunction with her/his primary care physician and the clinical team.

Case managers play a major role in facilitating access to needed services and in the ongoing co-ordination of care. The Massachusetts General Care Management Program design does not include a formal care package of care *per se*. That is, patients are referred to any and all services and supports they need. Care is delivered by providers beyond the Massachusetts General Care Management Program by Partners Healthcare affiliates and/or other agencies and institutions in the area. Patients must be eligible or qualified for the type and level of care to which they are referred.^12^

Case managers supported and co-ordinated patient care in numerous ways: for example, they educated patients about health-care options and resources, as well as medication and treatment regimens in order to help patients (and families) make informed choices and increase adherence to the care plan. They assisted patients with self-management activities and in adopting new behaviours to avert acute exacerbations of chronic conditions, thus preventing or delaying hospitalisations. They reminded patients about physician appointments and diagnostic tests and arrange needed transportation assistance; they also determined the issues involved when appointments are missed and help with re-scheduling. Finally, they maintained ongoing communication with patients and providers to simplify access to timely information, assistance and support, including updates on changing patient needs, issues and circumstances.

#### Management of care transitions

Transitioning to a different care setting is a complex process presenting potential communications and co-ordination problems; it can also mark serious changes in the patient’s clinical status. Case managers played a key role in managing transitions of care for their patients throughout the acute illness episode. When patients were admitted to the emergency department or hospital, the case manager worked closely with the primary care physician to ensure that patients did not fall in the cracks and appropriate treatment, prompt discharge, and necessary follow-up care was provided.

Every time a patient moved from one setting to another (home to emergency department or emergency department to hospital), the case manager (and primary care physician) was alerted by email and pager. The case manager then followed a protocol to assess the situation and assist with the management of the patient as necessary. Several HiT tools are specifically designed to support the case management process during patient transitions.

When patients were discharged from the hospital, a centralised discharge case manager reconciled medications, made sure that post-discharge plans were implemented, and followed up with the Massachusetts General Care Management Program case manager about the discharged patient.

#### Specialised support resources

##### Mental health

Early on in the demonstration, Massachusetts General Care Management Program staff recognised that many of the complex patients enrolled in the programme experienced mental health problems. Subsequently, a mental health team, consisting of a director, clinical social worker, two psychiatric social workers and forensic specialist, was put together to address a wide range of psychiatric and substance abuse issues, including legal and guardianship concerns.

##### Medication management

Since there are many patients in the Massachusetts General Care Management Program population that take multiple medications, a pharmacist was involved in reviewing medication regimens to identify opportunities to reduce medications, adjust medication regimens or suggest alternative therapies.

##### End-of-life care

Case managers and primary care physicians involved in the programme received assistance from a nurse specialised in end-of-life issues. Education was provided on how to discuss end-of-life concerns with patients, as well as support patients in developing advance directives. Information was also provided on hospice services.

## Implementation and organisation

### Implementation

Planning for this type of demonstration actually began about 2.5 years before Massachusetts General Care Management Program was officially launched in August 2006. For example, a pilot was conducted at Mass General’s Revere Health Care Centre to study the impact of identifying patients irrespective of insurance coverage who would most likely be admitted to the hospital within 6–8 weeks and providing them with care management services. For a voluntary group of patients, the health centre-based case manager served as a “physician extender” by identifying gaps in care, arranging for needed services and helping with prescription medications. The programme evaluation showed that physicians were very satisfied with the pilot; they referred to the case manager as a “fairy godmother” [5].

In addition to being informed by this early practice-based care management experience, Massachusetts General Care Management Program programme leaders—with the strong support of the Mass General leadership—reached out to the major stakeholders (primary care physicians, case managers, nurses, psychiatrists and hospital managers) to help design the programme and build legitimacy and support. A cornerstone of this effort was the holding of four focus groups to obtain their perspectives on the proposed Massachusetts General Care Management Program model, as well as suggestions on useful interventions. One group session focused on the perspectives of representatives from social work, mental health, hospital-based case managers and Massachusetts General Physicians Organization practice leaders. Another round of group sessions was held with practices to explore with primary care physicians how Massachusetts General Care Management Program could add value to their practices. This constant input and feedback obtained from individuals throughout the continuum was also important in identifying potential obstacles and opportunities for improvement [7].

Even though some primary care physicians already worked with case managers, most physician practices expressed apprehension about the changes required to implement the Massachusetts General Care Management Program initiative. As a result, Massachusetts General Care Management Program programme leaders devised a two-pronged strategy to win the full buy-in of primary care physicians. First, a tailored approach was used to discuss the programme and its unique challenges for each group of practitioners on a practice by practice basis. Second, physician champions were identified in each practice with at least 10 Massachusetts General Care Management Program patients to smooth programme implementation. These physician champions identified ways to incorporate new practice-based case managers and also actively encouraged their colleagues to participate in the programme.^13^ Further work was also needed to assure practice-based nurses that practice-based care management was not a duplication of their role.

During the first 7 months of operations, Massachusetts General Care Management Program staff gained a better understanding of the characteristics and needs of their complex patients. For example, case managers found themselves having to spend more time than anticipated with patients afflicted with “out-of-control” medical problems; this challenged the depth and breadth of case management support. While it was found that some patients were initially sceptical about the programme, the relationships formed with case managers nonetheless soon became very close and positive. In addition, some problems were encountered with integrating PCBCM into smaller practices which seemed less equipped than larger practices in implementing the required procedures.

Overall, however, the biggest obstacle Mass General faced in implementing its Massachusetts General Care Management Program was the development of needed infrastructure. Given the short time frame devoted to programme delivery, the hiring of case management staff lagged and the information systems were not fully set up to manage the demonstration. Consequently, patient enrolment took longer, especially at practices without an assigned case manager.

### Organisation and governance

Massachusetts General Care Management Program is a collaboration between Mass General and Massachusetts General Physicians Organization. The programme does not constitute its own organisation but rather operates within the highly matrixed organisational and managerial environment of Mass General and Massachusetts General Physicians Organization. In this context, programmes are frequently developed and operated with multiple and crisscrossing command structures. The Massachusetts General Care Management Program as such is ultimately accountable to the governing bodies of the two partner organisations.

The programme is jointly led by the Senior Vice President for the MGH/Massachusetts General Physicians Organization Center for Quality and Safety (Gregg Meyer, MD, MSC) the Medical Director of the Massachusetts General Physicians Organization (Tim Ferris, MD, MPH).

The Massachusetts General Care Management Program case management team is housed in Mass General’s Department of Case Management. However, Massachusetts General Physicians Organization provides overall programme administration and the underlying support structure for delivering integrated care management under the Massachusetts General Care Management Program.

The programme’s core management team consists of the Medical Director (Eric Weil, MD), Case Management Supervisor (Joanne Kaufman, RN, MPA) and Project Manager (Mary Neagle, MSW). The roles of these core team members are as follows:

- The Medical Director, a practicing internist with expertise in systems improvement methods, acted as the overall project facilitator. He engaged in ongoing communication with physicians, case managers and other staff; solicited their input; and, built consensus on key programme issues. He also led weekly case reviews and operations meetings, and served as the liaison between the Massachusetts General Care Management Program and physician practices.
- The Case Management Supervisor, who has extensive experience as a Case Management Department administrator and considerable clinical management experience in outpatient, inpatient and community programmes, was responsible for hiring, training and supervising programme case managers.
- The Project Manager, a social worker with extensive experience in planning, programme development and project management, was responsible for making sure the programme ran smoothly.

These three core management team members shared responsibility for monitoring and evaluating programme performance, overseeing the continuous quality improvement process, identifying and resolving operational issues, managing the programme budget, and maintaining a learning organisation. They met frequently to review data, discuss operational issues, troubleshoot problems, brainstorm ideas for improvement and address budgetary issues [5].

### Context

As was discussed in the Background section in Part I of this case study, Massachusetts General Care Management Program is a site in the federal government’s Care Management for High Cost Beneficiaries demonstration. Mass General and Massachusetts General Physicians Organization had always worked closely together in supporting medical management activities for their patients. Both organisations also shared a larger vision of a restructured primary care system capable of managing complex chronic illness and also making more efficient use of health-care resources, including the hospital. Involvement in the Centers for Medicare and Medicaid demonstration provided both organisations with the flexibility needed to formally test a bundle of care management interventions known to be effective in managing high-cost, high-risk Medicare beneficiaries. A joint proposal was submitted to Centers for Medicare and Medicaid in 2004 in response to the agency’s request for proposals (RFP); it was selected in 2005 as one of six demonstration projects nationwide.

The Massachusetts General Care Management Program serves Medicare fee-for-service beneficiaries, i.e., individuals who are not enrolled in capitated Medicare Advantage health plans. Patients also had to meet the programme’s other inclusion (eligibility) criteria. Centers for Medicare and Medicaid funding only covers the costs of the core interventions and support services provided by the Massachusetts General Care Management Program to patients voluntarily enrolled in the programme as part of the Care Management for High Cost Beneficiaries demonstration; these core interventions and support services were described earlier in the case study. All other care received by the patient, whether in the hospital, outpatient, home health care, rehabilitation or skilled nursing facility setting, are directly reimbursed to the respective provider through the traditional Medicare fee-for-service programme provided the benefits are covered. The patient’s participation in the Massachusetts General Care Management Program does not change the scope, duration or amount of Medicare fee-for-service benefits received.

Under the terms of its contract with Centers for Medicare and Medicaid, Massachusetts General Care Management Program received a negotiated per-beneficiary-per-month (PBPM) administrative fee of $120 to operate the project for the duration of the demonstration.^14^ At the end of the 3-year period, Mass General was obligated to achieve a 5% savings in Medicare-covered direct service payments among the intervention group (whether or not participating in the Massachusetts General Care Management Program) as compared to the comparison group, as well as cover all administrative fees collected from Centers for Medicare and Medicaid for programme operations.^15^ Moreover, if net savings were beyond the 5% requirement, Mass General had an opportunity to share a portion of these savings with Centers for Medicare and Medicaid.^16^

## Impact and sustainability

### Evidence of impact

Research Triangle Institute International, a private research and evaluation contractor, was engaged by Centers for Medicare and Medicaid to evaluate the Care Management for High Cost Beneficiaries demonstration, including Massachusetts General Care Management Program. In addition to conducting a beneficiary survey of the programme’s intervention and comparison populations, the evaluator made two rounds of site visits. The first visit took place at the close of the outreach period, and the second was held 2 years thereafter. The comprehensive evaluation focused on various aspects of programme start-up, evolution and operations. Detailed qualitative and quantitative data were collected through in-person and telephone interviews and secondary sources, e.g., programme monitoring reports. Research Triangle Institute International also conducted an assessment of patient satisfaction which examined the programme’s impact on enrolee care experience, self-management and physical and mental health functioning.

The Final Report, which was issued in September 2010, presents findings on process and outcomes for Mass General’s Massachusetts General Care Management Program based on the full 3 years of operation with its original population and 2 years with its refresh population [6]. Overall, the programme is considered a major success by Massachusetts General Care Management Program with 7% net savings achieved among participants [8].

The eight key findings from the Final Report on the first phase of the project are briefly summarised below:

*Key Finding #1 (participation levels)*: The Massachusetts General Care Management Program was very successful in recruiting participants, i.e., 88% of the original population and 84% of the refresh population. Patients who enrolled were broadly representative of the intervention population, and there were few significant differences between participants and nonparticipants in both the original and refresh groups.

*Key Finding #2 (targeting)*: Massachusetts General Care Management Program successfully targeted Medicare beneficiaries with high rates of acute care utilization and, therefore, at high risk of rehospitalisation.

Key Finding #3 (*patient Satisfaction, care experience, physical functioning, patient coping, self-care and mental health functioning*): Patients enrolled in the Massachusetts General Care Management Program were satisfied with their health-care team, and also experienced improved physical functioning. However, no other statistically significant outcomes were found with respect to patients’ coping with chronic conditions, self-care or mental health status.

*Key Finding #4 (quality of primary care physician work life)*: Participating primary care physicians reported overall satisfaction with the quality of medical practice and the quality of care being provided to their patients as a result of the Massachusetts General Care Management Program.

*Key Finding #5 (rate of compliance in quality of care process measures)*: No evidence was found in the original or refresh populations of systematic improvement in four selected process measures: influenza vaccination; low-density lipoprotein cholesterol (LDL-C) testing for patients with diabetes and ischemic vascular disease (IVD); and rate of annual HbA1c for patients with diabetes.^17^

*Key Finding #6 (impact on acute care hospitalisations, 90-day readmissions, emergency department visits, and end-of-life/hospice care)*: The Massachusetts General Care Management Program was successful in substantially reducing the rate of all-cause and ambulatory sensitive condition (ACSC) hospitalisations^18^ and emergency department visits in both the original and refresh patient populations. However, the programme did not reduce the rate of readmissions.

No statistically significant differences were found between the intervention and comparison populations in either the original or refresh groups with respect to the preparation of new advance medical directives or in the use of the Medicare hospice benefit as measured by mean and median days spent in Medicare hospice care.

The programme’s lack of impact on 90-day readmissions during phase one is a singularly disappointing result. Hospital readmissions are difficult for patients and their families and cost Medicare more than $17 billion each year [9]. In general, the evaluator observed a pattern of similar increases in both the intervention and comparison groups with an all-cause and ambulatory sensitive condition same-cause readmission or the rate of readmission per 1,000 beneficiaries during the early stage of the demonstration (7–18 months) and the last 12 months of the demonstration [6].

*Key Finding #7 (impact on mortality rate)*: Over the 3-year demonstration period, the Massachusetts General Care Management Program was successful in reducing the mortality rate of the original population as compared to the comparisons after adjusting for baseline characteristics.

*Key Finding #8 (impact on costs)*: The Massachusetts General Care Management Program achieved substantial, statistically significant savings over fee-for-service patients being served in the traditional Medicare system. Looking at return on investment (ROI), every dollar of programme fees invested by Centers for Medicare and Medicaid in the demonstration yielded $2.65 in savings on Medicare health-care services for the original intervention group; $3.35 for the refresh group.

### Sustainability and spread

Massachusetts General Care Management Program was one of only three sites (out of a total of six) participating in the Care Management for High Cost Beneficiaries demonstration initiative to achieve a positive impact on Medicare beneficiaries and meet or exceed cost savings requirements. As a result, Centers for Medicare and Medicaid announced in January 2009 that the programme would be the extended for three more years. During the second 3-year phase, which ended on 31 July 2012, the programme was expanded beyond Mass General to eligible patients to two additional hospitals in the Partners Healthcare system.^19^

The first phase of the Mass General programme has, for the most part, produced very encouraging results with respect to the impact of intensive, patient-centred practice-based care management on quality of care and costs for high-risk Medicare patients in the fee-for-service system. Enormous lessons can, therefore, be learned from this experience about how to best design, develop, implement and operate such programmes. Nonetheless, as the evaluation found, there are some shortcomings associated with the Massachusetts General Care Management Program, particularly its inability to reduce rates of hospital readmission. This is an especially disappointing finding in light of the programme’s major focus on preventing hospitalisations and re-hospitalisations, and the great amount of time and effort spent by case managers with their most at-risk patients.

The second and last phase has addressed these issues albeit within a greatly expanded programme. There have also been several modifications: First, eligible patients being seen by primary care physicians affiliated with Brigham and Women’s Hospital (an academic medical centre) and North Shore Medical Center (a community hospital) were are now able to participate along with those at Mass General. By the end of the demonstration, total enrolment reached approximately 8,300 patients across the three sites. Second, the programme’s scope of engagement was extended to post-acute (nursing home and rehabilitation) providers, since it is recognised that skilled nursing facilities or skilled nursing facilities in particular are associated with hospital readmissions. To enhance pre- and post-discharge connections with these non-acute institutions, Centers for Medicare and Medicaid approved a waiver of the so-called 72-hour rule.^20^ In addition, the Massachusetts General Care Management Program embarked on several other activities to enhance the management of care transitions. They created a network of post-acute providers, implemented a weekly “telerounding” system to report on patients in post-acute facilities, actively engaged case managers in planning for post-acute care and used standardised protocols to guide care transitions. Third, the programme increased its presence in the emergency department at the original Mass General Hospital site by adding a full-time equivalent (FTE) case manager to specifically assist with high-risk patients.^21^

The second phase was a period of stabilisation. Once institutionalised, the programme was no longer viewed internally as a pilot. Nonetheless, some new challenges emerged. At one of the hospitals, roughly half the size of Mass General Hospital, only six case managers were hired as opposed to the 12 originally assigned at Mass General. This created a coverage problem which appears to have affected the site’s performance. At the community hospital site, which included several private practices, case managers ended up being tied less closely with the primary care physicians than envisaged in the original model; this too has apparently been responsible for less than optimum results. Evaluation of the second phase will not be available from Research Triangle Institute International June 2013.

Partners Healthcare was selected by Centers for Medicare and Medicaid in December 2011 as one of 37 Pioneer accountable care organisations nationally,^22^ and began operations in January 2012. The Massachusetts General Care Management Program model supplies the essential framework—staffing structure, practice standards and protocols, collaborative approach, and information technology tools—for managing the complex medical needs of high-risk patients who will receive care in the new Partners Healthcare Pioneer accountable care organisation.

The Massachusetts General Care Management Program experience will be helpful to other organisations contemplating the development of a Pioneer accountable care organisation. However, two challenges come to mind with respect to the sustainability and spread of the original stand-alone model to other hospitals and primary care providers in the traditional Medicare fee-for-service system: first, it is unclear whether the programme is possible without the Centers for Medicare and Medicaid investment and incentives provided under the CMHBC demonstration. At present, the initiative is not a permanent part of the Medicare programme. Second, there is a question about the ability to translate the Massachusetts General Care Management Program model to the community hospital setting. Community hospitals have fewer resources than can be drawn upon by large and sophisticated academic medical centres like Mass General and the other teaching affiliates operating within the integrated system of Partners Healthcare. Finally, it is apparent that the Massachusetts General Care Management Program programme is not optimally designed to work in a solo practice environment; problems encountered with the rollout of the demonstration’s second phase call attention to this weakness.

With respect to other countries, there are many translatable elements in the Massachusetts General Care Management Program model that can theoretically “fit” in health systems which are considering strategies to address the challenges of similar high-risk, high-cost elderly populations.

## Barriers and facilitators to effective implementation^23^

### Systemic and contextual factors

There were a number of unique systemic and contextual factors that had an impact on the ability of the Massachusetts General Care Management Program to operate effectively:

As members of Partners Healthcare, a major integrated system of care in Boston and the surrounding region, Mass General and Massachusetts General Physicians Organization have long worked closely together to improve the co-ordination and quality of care for its patients. Both organisations also shared a public commitment to creating new knowledge about medical management, as well as providing national leadership to creatively address challenges like those posed by high-risk, high-cost Medicare patients. Finally, there was common concern about the future of primary care and the need for its revitalisation.

Both organisations recognised the need to do a better job with the care of their sickest and most vulnerable elderly patients. Mass General was losing money on these patients every time they were admitted to the hospital under Medicare’s Diagnosis-Related Group (DRG) prospective payment system. Furthermore, primary care physicians and other providers were spending a disproportionate amount of time and effort with these patients without improving health outcomes [5].

The growing focus on care co-ordination in the public and private system sectors underscored the need to find a workable solution for patients being cared for in the fragmented, fee-for-service health-care system.

Involvement in the Care Management for High Cost Beneficiaries demonstration made it possible for Mass General and Massachusetts General Physicians Organization to formally establish and test a co-ordinated care design tailored to the needs of high-cost, fee-for-service Medicare beneficiaries. Although the programme has been extended by Centers for Medicare and Medicaid, permanent Medicare funding will not be available to support its continuation after completion of the second phase unless a major change in federal policy is forthcoming.

### Organisational factors

A number of organisational factors and processes were associated with the ability of team members in the Mass General programme to effectively deliver services:

The design, development, implementation and operation of the Massachusetts General Care Management Program took place within an integrated health-care system where partner organisations shared a common mission, values and culture and management was used to working across organisational boundaries in a highly matrixed environment.

As a large academic medical centre operating within an integrated health-care delivery organisation, Mass General and Massachusetts General Physicians Organization were able to leverage enormous resources and capabilities to support the Massachusetts General Care Management Program. In addition to a large hospital-based case management department, a wide range of health-care services, and network of member primary care physicians, resources included access to a sophisticated electronic medical record system and integrated cost accounting system capable of providing real-time patient care data and analysis. Finally, extensive system-wide experience existed prior to the demonstration in designing, demonstrating and evaluating care management programmes of various kinds.

The programme was backed up by strong leadership on the system’s most senior levels; these individuals were directly involved in spearheading the demonstration. At the project level, day-to-day leadership was vested in a small, highly effective management team—medical director, case management supervisor and project manager—to run and constantly improve the programme.

From the inception of the demonstration, considerable emphasis was placed on building and maintaining legitimacy and support for the programme among stakeholders at all levels in order to develop a common vested interest in making the initiative successful, as well as overcome any concern or hesitation about the changes needed and their implementation. This was especially important with respect to the critical involvement of primary care physicians. One of the reasons why the programme was organised around the traditional fee-for-service system was that physicians were worried about placing their practices at financial risk with global or capitated payment models.

The case managers hired were very experienced and highly skilled in managing the clinical needs of patients with complex chronic illnesses, as well as in developing trusting relationships with patients and physicians. In addition to having clear professional roles and responsibilities, case managers performed their many tasks using standardised, but flexible workflows which enabled them to meet the personalised needs of their patients while facilitating programme efficiency at the same time. In addition, case managers were encouraged to keep at the top of their knowledge by attending required case conferences and continuing education classes.

A comprehensive information technology system supported the entire programme. Case managers had unrestricted access to the hospital’s electronic medical record, a specialised case management system, notification tools, population lists and email to facilitate workflow, obtain real-time patient care data and streamline clinical documentation. Information technology support was also instrumental in saving time and allowing case managers to carry larger caseloads.

At the core of the Massachusetts General Care Management Program was a commitment to organisational learning which was focused on how to improve quality of care and reduce health-care costs. The management team engaged with case managers at weekly meetings to examine the programme’s impact on measureable outcomes (emergency department visits, hospitalisations, readmissions, etc.) and devise solutions for identified problems using Deming’s Plan-Do-Study-Act (PDSA) cycle. Weekly “virtual rounds” reports were also required from case managers to highlight successes and challenges, as well as assist with the identification of new opportunities for systematic improvement. Further, the programme’s open culture encouraged the staff to solve problems on their own.

Massachusetts General Care Management Program programme managers held case managers accountable for their performance. Using data from the case management record system, the management team was able to track case management activity over time and compare the performance of individual case managers. This process was also useful in identifying workflow inefficiencies and helping with staff improvement.

Finally, the Massachusetts General Care Management Program was operated under tight financial control given the $120 per member per month administrative fee that Mass General negotiated with Centers for Medicare and Medicaid. To keep costs within budget, extensive resources were leveraged from throughout the Partners Healthcare system. Perhaps as important, the project manager paid careful attention to keeping the programme on a sound financial footing.

### Operational and service delivery factors

Lastly, what operational and service delivery factors are associated with making co-ordination at the patient level so effective in Massachusetts General Care Management Program?

As was discussed earlier, the Massachusetts General Care Management Program model followed a largely evidence-based design that included the following six main characteristics:

- Careful targeting of a complex patent population with pro-active case finding using a combination of claims data analysis and risk prediction methods.
- Highly trained and experienced nurse case managers embedded in the physician practice setting.
- Single point of health risk appraisal, comprehensive assessment, care planning and co-ordinated access to services using evidence-based support tools.
- Intensive case manager–patient relationships with high levels of face-to-face contact.
- Active communication systems between patients and providers with the case manager serving as the hub.
- Support provided to patients to help them manage chronic conditions and medication regimens, prevent avoidable emergency department visits and hospitalisations, address mental health concerns and deal with end-of-life issues.

From the provider’s perspective, the model’s most powerful innovation is the co-location of case managers with primary care physicians in the practice setting. Rather than being separate and apart from the delivery of primary care, case managers work in close collaboration with primary care physicians and other members of the clinical team at the point of service. This is an important enabler of co-ordinated care.

From the patient’s perspective, the close, trusting, one-on-one relationships developed by case managers appeared to help patients stay informed, connected and satisfied with their care experience. These are important quality of life outcomes in their own right. Moreover, such relationships enabled the case manager to keep on top of the patient’s condition, identify changing needs, spot warning signs, maintain open communications and facilitate co-ordination and continuity of care.

We know from experience that family involvement in patients’ care leads to greater success of care management programmes [10]. What is not so clear is the role that family carers played in the Massachusetts General Care Management Program or how they were supported.

## Conclusions

The success of phase one of the Massachusetts General Care Management Program has attracted enormous attention in the United States. While the second phase of the demonstration has finally ended, it remains to be seen from the formal evaluation expected in June 2013 whether it was effective in addressing unresolved issues from the first phase and also if the model proved workable within the context of a community hospital setting.

The Massachusetts General Care Management Program model does not rest upon a “whole systems” approach. It is an excellent example of how an innovative care co-ordination programme can be implemented in an existing health-care organisation without making fundamental changes in its underlying structure or the way in which direct patient care services are paid for. Nonetheless, it should be recognised that Mass General is no ordinary health-care provider. The programme benefited greatly from the existing integration of hospital and physician services, universal use of an electronic medical record, the availability of advanced clinical and administrative information systems, an extensive network of primary care physicians and a wide range of acute and chronic care services [4]. These characteristics may limit the model’s generalisability in the United States, especially in the absence of the kind of incentives found in the original Medicare Care Management for High Cost Beneficiaries demonstration in which the Mass General pilot took place.

The Mass General experience described in this case study, however, goes beyond the Massachusetts General Care Management Program model. In a world where accountable care organisations are being touted as the future of American health care, it is probably safe to say that Partners Healthcare would not have agreed to become a Pioneer accountable care organisation without the apparent success of this innovative integrated care programme.

Finally, the core components and interventions found in the original Massachusetts General Care Management Program demonstration should be helpful to health-care systems in other countries which are in search of evidence-based care co-ordination strategies to address the challenges presented by high-risk, high-cost elderly patients.

## Figures and Tables

**Table 1.**
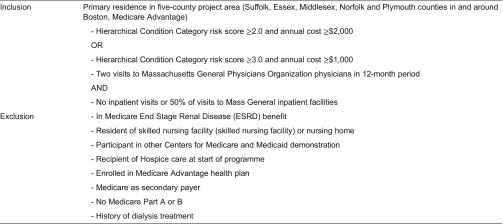
Inclusion (eligibility) and exclusion criteria

Source: Ref. [4].

**Table 2.**
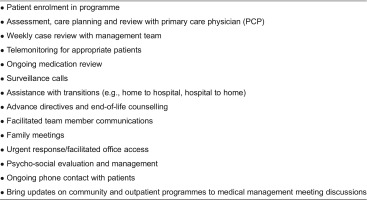
Major case manager roles, responsibilities and activities

Source: Refs. [4] and [5].

**Table 3.**
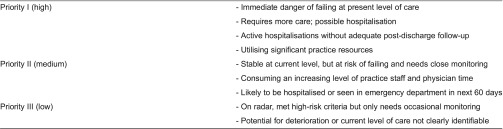
Patient risk stratification

Source: Ref. [5].

**Table 4.**
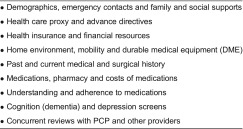
Comprehensive patient assessment domains

Source: Ref. [5].

## Notes

^1^​ Fee-for-service (FFS) is a health-care payment model wherein services are unbundled and reimbursed separately. FFS creates a potential conflict of interest and is known to incentivise overutilisation in terms of service volume and cost.

^2^​ Medical home, also known as the patient-centred medical home, is an important new model of physician-led, team-based comprehensive primary care in which care co-ordination and innovative payment methods to enhance quality outcomes and cost-effectiveness are essential components.

^3^​ Accountable care organisations or ACOs are networks of physicians, hospitals and other health-care providers that voluntarily come together and are held accountable for the quality and cost of the entire continuum of care delivered to a group of patients. While ACOs are being developed in the private sector, the Centers for Medicare and Medicaid Services (CMS) is authorised under the Affordable Care Act to contract with various ACO entities under the Medicare program.

^4^​ Medicare is the national social insurance program administered by the federal government that guarantees access to health insurance for Americans aged 65 and over, people with disabilities, and people with End Stage Renal Disease. Established by the Title XVIII of the Social Security Act in 1965, the program covers hospital benefits (Part A) and outpatient medical services (Part B). A majority of Medicare beneficiaries (76%) receive their care in the traditional FFS system; the rest (24%) are voluntarily enrolled in Medicare Advantage health plans.

^5^​ CMS is an agency of the U.S. Department of Health and Human Services; it is responsible for running the Medicare programme.

^6^​ P4P is a “value-based purchasing” arrangement designed to reward health-care providers for meeting certain quality and efficiency goals, e.g., patient management improvements, better health outcomes and the avoidance of unnecessary costs.

^7^​ Several inclusion (eligibility) criteria listed in Table 1 (e.g., two visits to MGPO physicians for a selected group of outpatient and emergency department procedures, and a majority of inpatient visits made to Mass General facilities) are measures of patient loyalty. CMP sponsors strongly believe that care management interventions have maximum impact on “loyal” patients who are more prone to active engagement with the programme and their case manager. The other advantage of focusing on a loyal patient base is that access to clinical and cost data for program management, clinical, quality and evaluation purposes is greatly enhanced.

^8^​ This patient pool was refreshed during the first phase to take into account patients and primary care physicians lost to the program. CMS limited the size of this cohort to 30% of the original eligible intervention group, or 785 patients, who were randomly selected from an additional pool of 1,870 identified using the same selection procedures as initially.

^9^​ In addition to being located in a physician practice, each case manager “floated” to one or two small practices with five or fewer patients.

^10^​ Mass customisation is an operations management method which combines standardised production techniques with the flexibility of customisation and personalisation in order to provide high-quality goods and services to consumers at lower unit costs.

^11^​ As the number of patients in the demonstration grew, the equivalent of two additional community resource specialists were added to the team. These professionals supported referrals and linkages to community services such as transportation, meals-on-wheels, and housing assistance available from federal-, state- and locally funded agencies in the project area.

^12^​ This also implies that referred services must be covered by Medicare or some other source of payment.

^13^​ The most common concern voiced by primary care physicians in the early stage of the program was their frustration with not being able to include additional patients.

^14^​ Other than for the core programme activities, the fee did not cover the costs of health information systems, data and financial analytics, and individual practice overhead.

^15^​ CMP was allowed to “refresh” its population to offset enrolee attrition primarily due to death. With respect to the refresh group, Mass General was at financial risk for administrative fees received from CMS plus an additional 2.5%; not 5% as in the original population.

^16^​ For example, shared savings in the >5–10% range would be paid 100% to Mass General; savings in the >10–20% range would be shared equally between Mass General (50%) and CMS (50%).

^17^​ From the beginning, Mass General CMP's leadership felt that these measures were, for the most part, not appropriate benchmarks for measuring the process quality for care of very sick and chronic patients enrolled in a co-ordinated care program. In addition to ED and hospital utilization rates, they offered measures such as follow-ups on patient initiated calls, etc.

^18^​ The rate of all-cause and ACSC hospitalizations increased in the original intervention and comparison groups between the baseline and both demonstration periods (7–18 months and the last 12 months). During 7–18 months of the demonstration, a substantially lower difference-in-differences rate of growth was observed within the intervention group. The rate of all-cause hospitalizations increased by 48% in the comparison group vs. only 13% in the comparison population during the same period [6]].

^19^​ While the second phase of the Mass General CMP officially ended at the end of July 2012, it ceased receiving Medicare PMPM payments on 31 December 2011.

^20^​ This rule, which tends to confound and frustrate, requires patients to spend at least 72 hours in the hospital for medically appropriate treatment and assessment before Medicare will pay for qualified follow-up care in an SNF. Failure to meet this regulation is a major obstacle to the provision of integrated care and also means that the patient must pay for care in the SNF on a private basis.

^21^​ As part of the aforementioned waiver of the 72-hour rule, the case manager was authorised in particular to admit a patient directly from the ED to an SNF without the required prior hospital stay.

^22^​ The “Pioneer” ACO model enables health-care organisations and providers that are already experienced in care co-ordination across settings to move rapidly from FFS based on a shared savings formula to a population-based payment approach under Medicare [12]].

^23^​ This section is largely based on an excellent study by Kamo (2010) which examined the critical factors which contributed to the success of the Mass General CMP. The report examined four main domains: organisational strategy, programme design, programme implementation (front end and back end) and challenges.
